# Outbreaks Caused by New Variants of *Vibrio cholerae* O1 El Tor, India

**DOI:** 10.3201/eid1502.080943

**Published:** 2009-02

**Authors:** Neelam Taneja, Arti Mishra, Garima Sangar, Gagandeep Singh, Meera Sharma

**Affiliations:** Postgraduate Institute of Medical Education and Research (PGIMER), Chandigarh, India

**Keywords:** Cholera, new variant V. cholerae O1, India, letter

**To the Editor:** *Vibrio cholerae* O1, the causative agent of cholera, has 2 biotypes (classical and El Tor), which have traditionally been distinguished by phenotypic tests and by genetic differences in the major toxin-coregulated pilus (TCP) gene, the *tcpA* allele of the TCP cluster (*1*), the *rstR* region (regulatory region for phage lysogeny) of CTX phages (*2*), the type of cholera toxin (CT) produced, and the infection pattern of the disease they cause. However, 3 variants of the El Tor biotype have been described recently: Matlab (a place in Bangladesh) variants in 2002 (*3*), which could not be biotyped because they have a mixture of both classical and El Tor (*4*), Mozambique variant in 2004–2005, which has a typical El Tor genome but a tandem repeat of the classical CTX prophage in the small chromosome (*5*), and the altered El Tor type (a typical El Tor biotype and an El Tor CTX prophage that produces CT of the classical type) predominant in Bangladesh since 2001 (*6*). Hybrid vibrios have also been described in other regions of Asia and Africa (*7*).

CT, encoded by the *ctxA* and *ctxB* genes, is the principal toxin produced by *V. cholerae* O1 and O139. Methods for differentiating the biotype-specific CT-B subunit of *V. cholerae* O1 include sequencing the *ctxB* gene, performing an ELISA wth a monoclonal antibody specific to the classical or El Tor CT, or by using a mismatch amplification mutation assay (MAMA)–PCR to distinguish between 2 kinds of *ctxB* genes. This assay detects sequence polymorphisms based on nt position 203 of the *ctxB* gene (*8*).

In Punjab and Haryana states of northern India, during July–September 2007, 6 clusters of cholera outbreak were identified. A total of 745 case-patients were admitted to local government hospitals; the cholera attack rate was 183/1,000 population. Four deaths were reported (case-fatality rate 0.5%). The number of cases per cluster varied from 15 to 400, and adults were primarily affected (74%); 20% of patients had severe dehydration. *V. cholerae* O1 Ogawa was confirmed from stool cultures by using standard isolation, biochemical, and serotyping methods. Twenty-six isolates were phenotypically and genotypically characterized according to biotype. Phenotypic characterization included the Voges-Proskauer reaction, polymyxin B (50 U) susceptibility, chick cell agglutination, and sheep erythrocyte hemolysis; all isolates were confirmed as El Tor biotype. Genotypic characterization included PCR assays for *ctxA* and *tcpA* (*2*), *rstR* (*3*), and *ctxB* (*8*). All strains were toxigenic because each carried the *ctxA* gene. All strains also carried El Tor–specific *tcp*A (472 bp) and *rst*R genes (500 bp*).* MAMA-PCR showed the *ctxB* gene of both El Tor and the classical type in 21 (80%) of 26 isolates tested.

Similarly, we also tested 20 available isolates from the 2002 outbreak and 4 and 19 sporadic isolates from 2003 and 2004, respectively; all were phenotypically and genotypically confirmed as El Tor and had only *ctxB* of the El Tor type. Of 53 water samples tested during the 2007 outbreak, 4 grew *V. cholerae*. Three samples were confirmed to be non-O1, non-O139 strains. Only 1 isolate was *V. cholerae* O1, which was positive for tcp, *ctxA*, and *ctxB* of both classical and El Tor types (Table).

**Table:** Phenotypic and genotypic traits of *Vibrio cholerae* O1 clinical strains isolated from northern India, 2002–2007*

Year of isolation or type of strain	Phenotypic tests					PCR amplicons		
	VP	Polymyxin B (50 U) susceptibility	CCA	Sheep erythrocyte hemolysis		*tcpA*	*ctxB* (MAMA-PCR)	*rstR*
2002 (n = 20)	+	Resistant	+	+		E	E	E
2003 (n = 4)	+	Resistant	+	+		E	E	E
2004 (n = 19)	+	Resistant	+	+		E	E	E
2007 (n = 26)	+	Resistant	+	+		E	E + C (n = 21)†	E
Environmental (n = 1)	+	Resistant	+	+		E	E + C	E
Classical 569B	–	Sensitive	–	–		C	C	C
El Tor N16961	+	Resistant	+	+		E	E	E
Hybrid NICED, India	+	Resistant	+	+		E	C	E + C

*VP, Voges-Proskauer; CCA, chick cell agglutination; MAMA, mismatch amplification mutation assay; +, positive; –, negative; E, El Tor type; C, classical type; NICED, National Institute of Cholera and Enteric Diseases, Kolkata, India. †Remainder had *ctxB* of El Tor type.

During the cholera outbreak of 2002 in Chandigarh (*9*), only 1 death was reported (case-fatality rate, <0.01%); the attack rate was 20/1,000, 58.6% were children, and only 10% had severe dehydration. Before the most recent outbreak, the affected regions of Panjab and Haryana (Ambala, Nurpur, Kurali, Mohali, Panchkula, and Raili) had been free of cholera outbreaks since 1994, though sporadic cases had been reported. The 4 deaths from cholera in 2007, along with adult preponderance, high attack rate, more severe illness, and 6 different clusters, point towards a change in the disease’s epidemiology. This change may be related to circulation of the hybrid vibrios in this region. In Bangladesh, all strains of *V. cholerae* O1 examined since 2001 belong to the altered El Tor type (*6*), which produces CT of the classical type. This altered type has replaced the seventh pandemic strain of the El Tor biotype that produced CT of the El Tor type, which indicates that a cryptic change has occurred in the seventh pandemic El Tor biotype strains of *V. cholerae* O1.

Newly emerged variants from Bangladesh (*8*) have the genetic makeup of El Tor with *ctxB* gene of only classical, whereas our strains are unique in having *ctxB* of both the classical and El Tor biotypes. Our strains appear to be different from the Mozambique variant *V. cholerae* O1 (*10*), which has *rstR* of the classical type, in that our strains have *rstR* of only the El Tor type. Of 5 Matlab variants analyzed with MAMA-PCR, 3 had classical *ctxB* and 2 had El Tor type. Our study highlights the different genetic recombinations possible in *V. cholerae* and the epidemiologic role of these recombinations.
